# Effects of VEGFR1^+^ hematopoietic progenitor cells on pre-metastatic niche formation and in vivo metastasis of breast cancer cells

**DOI:** 10.1007/s00432-018-2802-6

**Published:** 2018-11-27

**Authors:** Du Meng, Min Meng, Anqi Luo, Xin Jing, Guanying Wang, Shangke Huang, Minna Luo, Shan Shao, Xinhan Zhao, Rui Liu

**Affiliations:** 1 Department of Oncology, First Affiliated Hospital of Medical School of Xi’an Jiaotong University, 277# Yanta west road, Xi’an, 710061 Shaanxi People’s Republic of China; 2 Department of Radiation Oncology, First Affiliated Hospital of Medical School of Xi’an Jiaotong University, Xi’an, 710061 Shaanxi People’s Republic of China; 3 Department of Oncology, Shandong Provincial Hospital Affiliated with Shandong University, Jinan, 250021 Shandong People’s Republic of China; 4 Department of Hematology, First Affiliated Hospital of Medical School of Xi’an Jiaotong University, Xi’an, 710061 Shaanxi People’s Republic of China

**Keywords:** VEGFR1, Hematopoietic progenitor cells, Lung metastasis, Pre-metastatic niche, Protein microarray

## Abstract

The pre-metastatic niche has been shown to play a critical role in tumor metastasis, and its formation is closely related to the tumor microenvironment. However, the underlying molecular mechanisms remain unclear. In the present study, we successfully established a mouse model of lung metastasis using luciferase-expressing MDA-MB-435s cells. In this model, recruitment of vascular endothelial growth factor receptor-1 (VEGFR1)^+^CD133^+^ hematopoietic progenitor cells (HPCs) was gradually increased in lung but gradually decreased after the formation of tumor colonies in lung. We also established a highly metastatic MDA-MB-435s (MDA-MB-435s-HM) cell line from the mouse model. Changes in protein profiles in different culture conditions were investigated by protein microarray analysis. The levels of CXC chemokine ligand 16, interleukin (IL)-2Rα, IL-2Rγ, matrix metalloproteinase (MMP)-1, MMP-9, platelet-derived growth factor receptor (PDGFR)-α, stromal cell-derived factor (SDF)-1α, transforming growth factor (TGF)-β, platelet endothelial cell adhesion molecule (PECAM)-1 and vascular endothelial (VE)-cadherin were significantly greater (> fivefold) in the culture medium from MDA-MB-435s-HM cells than in that from MDA-MB-435s cells. Moreover, the levels of MMP-9, PDGFR-α, and PECAM-1 were significantly greater in the co-culture medium of MDA-MB-435s-HM cells and CD133^+^ HPCs than in that from MDA-MB-435s-HM cells. Differentially expressed proteins were validated by enzyme-linked immunosorbent assay, and expression of their transcripts was confirmed by quantitative real-time polymerase chain reaction. Moreover, inhibition of MMP-9, PDGFR-α, and PECAM-1 by their specific inhibitors or antibodies significantly decreased cell migration, delayed lung metastasis, and decreased recruitment of VEGFR1^+^CD133^+^ HPCs into lung. Intra-hepatic growth of HPCs enhanced the invasive growth of MDA-MB-435s-HM cells in the liver. Our data indicate that VEGFR1^+^CD133^+^ HPCs contribute to lung metastasis.

**Electronic supplementary material:**

The online version of this article (10.1007/s00432-018-2802-6) contains supplementary material, which is available to authorized users.

## Introduction

Tumor metastasis is a dynamic and complex process involving a series of critical steps including epithelial-to-mesenchymal transition (EMT) of cancer cells, local invasion of primary tumor, tumor cell intravasation into the circulatory system, extravasation and seeding at the pre-metastatic niche, and finally invasion and growing in the secondary sites—most frequently in the lung and liver (Arvelo et al. 2016; Zingg et al. 2018). It has been demonstrated that tumor metastasis involves close collaboration of multiple cell types [cancer cells, immune cells, cancer-associated fibroblasts (CAFs) and bone marrow-derived cells (BMDCs)], inflammatory cytokines and chemokines, matrix metalloproteinases (MMPs), etc. (Dinesh and Rasool 2018; Shan et al. 2018).

Previous studies have achieved significant progress in understanding the molecular mechanisms of tumor metastasis (Pachmayr et al. 2017; Sadremomtaz et al. 2018). In 1889, Paget et al. first put forward the concept of “seed” and “soil” for tumor metastasis. Some studies had demonstrated that a distant organ as a site of tumor metastasis is not a passive receiver of circulating tumor cells but is actively selected and well prepared before the seeding of circulating tumor cells (Peinado et al. 2017). Chronic deregulation of cytokine expression and activity can result in tumor initiation, growth, and metastasis (Peinado et al. 2017; Itatani et al. 2016; Guan et al. 2018; Hou et al. 2017). Abnormal expression and secretion of cytokines not only remodels the tumor microenvironment, but also suppresses the capability of immune cells to kill tumor cells (Xiao et al. 2018; Caraci et al. 2018; Tan et al. 2018; Siveen et al. 2017). For example, transforming growth factor (TGF)-β1 is known as a tumor inhibitor during the early stage of different cancers, whereas it functions as a tumor promoter (promoting tumor invasion and metastasis) during the later stages (Heldin et al. 2017). Vascular endothelial growth factors (VEGFs) and their receptors (VEGFRs) play critical roles in angiogenesis during tumor growth and metastasis (Que et al. 2018; Shay et al. 2015). MMPs, known as physiologic scissors, are involved in extracellular matrix (ECM) degradation and tissue remodeling (Lee et al. 2018). In the tumor scenario, MMPs have been found to not only contribute to tumor invasion and metastasis but also to control cell growth, inflammation, and angiogenesis via multiple signaling pathways (Liu and Cao 2016; Mrozik et al. 2018; Giles et al. 2016). Moreover, recent studies found a new role for MMPs in building a favorable microenvironment for tumor metastasis (Itatani et al. 2016; Liu and Cao 2016).

Over the past decade, increasing evidence supports that circulating tumor cells from primary tumor can regulate the microenvironment of target organs and improve the seeding efficiency of incoming tumor cells via the formation of pre-metastatic niches (Clément-Demange et al. 2018; Xu et al. 2018). A pioneering study by Kaplan et al. defined the pre-metastatic niche and explored its temporal and functional relationship to metastasis. They demonstrated that non-malignant bone marrow (BM)-derived hematopoietic progenitor cells (HPCs) that express the vascular endothelial growth factor receptor-1 (VEGFR-1) can settle at distant sites before the arrival of metastatic tumor cells (Sahoo et al. 2018). They further clarified that tumor-specific factors can upregulate the expression of very late antigen-4 (VLA-4) ligand and fibronectin at distant pre-metastatic sites, recruit platelet-derived growth factor receptor (PDGFR)-expressing cells, and make the pre-metastatic niches ready for incoming tumor cells (Sahoo et al. 2018). A recent study by Zhang et al. also reported that CD133^+^ human umbilical HPCs might induce the proliferation or metastasis of colorectal cancer cells in the liver and impact the expression of their derived proteins such as mitogen-activated protein kinase 4 (MAPK4), stromal cell-derived factor-1 (SDF-1), MMP-9, calumenin, peripherin, leucine zipper, putative tumor suppressor 1, and guanidinoacetate methyltransferase (Lei et al. 2018). These studies have provided evidence that BM-derived HPCs can assist in preparing the pre-metastatic site for tumor metastasis. However, the molecular mechanisms responsible for the formation of pre-metastatic niche have not been elucidated and verified.

In the present study, we aimed to investigate the presence of the pre-metastatic niche during lung metastasis and to explore the potential molecular mechanisms in melanoma. To achieve these goals, we first generated a lung metastasis model in nude mice using the melanoma cell line MDA-MB-435 s expressing luciferase and detected the percentage of VEGFR1^+^CD133^+^ HPCs in the lung during the progression of lung metastasis. We further explored the changes in the expression profiles of cytokines and MMPs during communication between highly metastatic MDA-MB-435 s cells and CD133^+^ HPCs. The functions of differentially expressed cytokines and MMPs in lung metastasis were then validated by in vitro and in vivo studies.

## Materials and methods

### Cell culture and isolation of HPCs

The human MDA-MB-435s cell line, which was originally derived from pleural effusion of a ductal carcinoma patient but was recategorized as a melanoma cell line based on genetic background, was obtained from The Cell Bank of the Chinese Academy of Sciences (Shanghai, China) and cultured in 10% RPMI 1640 medium (Sigma-Aldrich, St Louis, MO, USA) supplemented with 10% fetal bovine serum (FBS, HyClone Laboratories, GE Healthcare Life Sciences, Logan, UT, USA) and 1% penicillin/streptomycin (Gibco, Life Technologies, Carlsbad, CA, USA) in a 37 °C incubator with 5% CO_2_. To establish the xenografted tumor model, MDA-MB-435s cells were infected with 20 multiplicity of infection (MOI) of pGC-FU-luciferase lentiviruses (Shanghai Jikai GeneChem Lit. Corp., China) supplemented with 5 µg/ml of polybrene overnight and selected with 5 µg/ml puromycin for 3 days to obtain a stable cell line.

This study was approved by the Ethics Committee of Xi’an Jiaotong University, and written consent was obtained from all women who donated umbilical cord blood (UCB) for this study. To isolate HPCs, human UCB samples were collected from healthy pregnant women after delivery with normal full-term pregnancy and without any complications. These samples were obtained in the Department of Obstetrics and Gynecology of the First Affiliated Hospital of Medical School of Xi’an Jiaotong University. UCB samples were collected in a 50-ml tube and processed within 6 h. The mononuclear cells isolated from UCB were suspended with Ficoll-Hypaque solution (Guangdong Weijia Biotechnology Company, Guangzhou, China) and centrifuged at 2500 rpm for 30 min. The cells were washed with phosphate-buffered saline (PBS) with 5% bovine serum albumin (BSA, Invitrogen, Life Technologies, Carlsbad CA, USA). The CD133^+^ enriched fraction was collected using the magnetic-activated cell sorting (MACS) method (Miltenyi Biotechnology Inc., Auburn, CA, USA). This procedure was repeated once to obtain a higher purity of CD133^+^ HPCs. The purification efficiency of CD133^+^ HPCs was examined using flow cytometry (FACSCalibur, Beckon Dickinson Biosciences, San Jose, CA, USA) using counterstaining with the monoclonal antibodies to CD133-PE (Miltenyi Biotechnology Inc.) and CD34-FITC (Miltenyi Biotechnology Inc.). The CD133^+^ HPCs were cultured in Iscove’s Modified Dulbecco’s medium (IMDM) supplemented with 15% FBS, stem cell factor (SCF, 50 ng/ml), thrombopoietin (10 ng/ml), Flt3 Ligand (Flt3-L, 50 ng/ml), and interleukin (IL)-6 (10 ng/ml) in a 37 °C humidified incubator with 5% CO_2_.

### Small molecular inhibitors of signaling pathways

Small molecular inhibitors, LY294002 (a specific inhibitor of PI3K/AKT pathway, cat #S1105), PD98059 (a specific inhibitor of MEK/ERK pathway, cat #S1177), CP690550 (a specific inhibitor of Jak/STAT pathway, cat #S2789), SB431542 (a specific inhibitor of TGFβ/Smad pathway, cat #S1067), and BIRB796 (a specific inhibitor of p38 MAPK pathway, cat #S1574) were purchased from Selleck Corporation (Houston, TX, USA). These agents were resolved in DMSO and diluted in DMEM without FBS for cell culture experiments.

### Establishment of lung metastasis model in nude mice

All animal studies were conducted in accordance with the UK Animals (Scientific Procedures) Act of 1986 and the associated guidelines (Wu et al. 2018). A total of 160 5-week-old female BALB/c nude mice weighing 18–20 g were purchased from Shanghai Silaike Experimental Animal Limited Corp. (China) and housed at the Experimental Animal Center of Medical School of Xi’an Jiaotong University. All animal experiments were approved by The Animal Care and Use Committee of Xi’an Jiaotong University. To evaluate the lung metastasis potential of MDA-MB-435s cells, one million MDA-MB-435s stable cells diluted in 100 µl of a mixture of RPMI1640 medium and Matrigel (1:1) were injected subcutaneously. A visible xenografted tumor node appeared at 12 days after injection. Lung metastasis of injected cells was monitored on days 2, 4, and 8 and every 4–6 days after the appearance of the tumor node. At each time point, 6 mice (*n* = 6) were intraperitoneally injected with 150 mg/kg fluorescein (Biotium Inc., Fremont, CA, USA) and anesthetized with isoflurane after 15 min. The bioluminescence of luciferase in the lung area was captured under an in vivo bioluminescence imaging system (Xenogen, USA). To examine the amount of VEGFR1^+^ HPCs in the lung, the xenografted mice (*n* = 6) were killed by cervical dislocation on days 2, 4, 8, 12, 18, 24, 36, 48, 68, 70, 74 and 80 after injection. The lung was perfused with 10–15 ml cold PBS from the right heart ventricle and then dissected and cut into small pieces in 1 ml saline. The cells were dispersed by repeated pipetting with a 5 ml syringe and then filtered with 200-mesh cell strainer. The isolated cells were used for flow cytometry of VEGFR1^+^ HPCs.

Mice were screened by a small animal in vivo imager to determine the intensity of luciferase activation following a method described by Li et al. (2017) and Liang et al. (2017). Then the mouse tissues were harvested, and photographs of tumor tissues were quantitatively analyzed using ImageJ software following a method reported by Shao et al. (2018) and Li et al. (2018) The results for luciferase intensity, tumor nodule area, and tumor nodule number are shown as mean ± standard deviation (SD).

### Flow cytometry

The cell suspension was diluted with PBS to a concentration of 1.0 × 10^6^ cells/ml, and 100 µl was added into each well of a 96-well plate (round bottom) in triplicate. Cells were then washed twice with cold PBS. The cells were labeled with monoclonal antibodies to anti-CD133-PE (eBioscience, Thermo Fisher Scientific, Waltham, MA, USA) and anti-VEGFR1-APC (R&D Systems, Minneapolis, MN, USA) at 4 °C in a refrigerator for 30 min and washed with PBS twice. The cells were resuspended in 500 µl PBS and analyzed using a FACSCalibur (Beckon Dickinson Biosciences).

### Isolation of highly metastatic MDA-MB-435s (MDA-MB-435s-HM) cells from the lung of the xenografted mouse model

The xenografted mouse model was generated as described above. At 74 days after injection (based on our results, the average time for lung metastasis of MDA-MB-435s cells was 68 days), the mice were killed and the lung was perfused, dissected, cut into small pieces in at least three volumes of 0.1% collagenase I solution containing 10 µg/ml DNase I, and then incubated at 37 °C for 1 h with gentle shaking. To terminate the digestion, 5 ml RPMI1640 medium containing 10% FBS was added and pipetted to dispense the cells. The cell suspension was centrifuged at 150*g* for 10 min, and the cell pellet was resuspended in 5 ml RPMI1640 medium, filtered with a 200-mesh cell strainer, and cultured in RPMI1640 complete medium. To reduce the contamination of fibroblasts, the cells grown in the flask were washed with PBS once and digested with 1 ml of 0.25% trypsin. The digestion reaction was observed under a microscope and terminated with 2 ml RPMI1640 complete medium when some cells became round and detached from the flask. Because fibroblasts detached from the flask first, the medium was discarded. The remaining cells were washed with PBS and digested with 1 ml of 0.25% trypsin. After complete digestion, 3 ml RPMI1640 complete medium were added and centrifuged at 120*g* for 3 min. The cells were washed with PBS and cultured in RPMI1640 complete medium. Because the number and shape of chromosomes differ between human and mouse, the purity of isolated human MDA-MB-435s cells from mouse lung was examined by chromosome staining using the conventional procedure (Supplemental Fig. 1). To obtain MDA-MB-435s-HM cells, the cells isolated in the first round were re-injected into nude mice and isolated from the lung as for the first round. The same xenografting procedure and tumor cell isolation from mouse lung were performed for six rounds, and the isolated cells from the sixth round of xenografted mice were regarded as MDA-MB-435s-HM cells and used for subsequent experiments.

### Protein microarray

Equal numbers of MDA-MB-435s cells, MDA-MB-435s-HM cells, CD133^+^ HPCs and co-cultured MDA-MB-435s-HM cells and CD133 + HPCs (50%:50%) were cultured in serum-free medium for 24 h, and the culture medium was collected for protein microarray. Protein microarray was carried out by Shanghai Wayen Biotechnology Corp. (China) following the standard protocols. Briefly, the protein chip (Cat. AAH-CYT-8, Raybiotech) was blocked by blocking buffer for 30 min at room temperature and then incubated with 100 µl of cell culture medium at 4 °C overnight. The chip was washed with 1 × wash buffer I and II twice and then incubated with detection antibody for 2 h at room temperature. The chip was washed with 1 × wash buffer II twice and incubated with Cy3 equivalent dye-conjugated streptavidin for 1 h at room temperature in darkness. After sufficient washing with 1 × wash buffer I and II, the chip was dried and scanned by an Axon GenePix 4000B microarray scanner (Molecular Devices LLC., Sunnyvale, CA, USA). The data were analyzed using GenePix Pro 6.0 software.

### Enzyme-linked immunosorbent assay (ELISA)

To verify the results of protein microarray analysis, the most differentially expressed proteins (> fivefold) were validated by ELISA. A high-binding 96-well plate was pre-coated with 100 µl of appropriate antibodies (1 µg/ml diluted in carbonate buffer) at 4 °C overnight. The following antibodies were used in this step: CXC chemokine ligand 16 (CXCL16, Invitrogen, cat #MA5-23869), IL-2Rα (Abcam, cat #ab46036), IL-2Rγ (R&D Systems, cat #YD1104), MMP-1 (Abcam, cat #ab100603), MMP-9 (Abcam, cat # ab100610), PDGFR-α (Abcam, cat #ab65258), SDF-1a (Abcam, cat #ab100637), TGF-β (Abcam, cat #ab100647), platelet endothelial cell adhesion molecule (PECAM)-1 (Abcam, cat #ab190814), and vascular endothelial (VE)-cadherin (Abcam, cat #ab210968). After washing with PBST twice, the plate was blocked by blocking solution for 1 h at room temperature and then incubated with 100 µl of the cell medium (in duplicate) used for protein microarray for 1 h at 37 °C. The plate was washed with PBST and incubated with diluted detection antibody for 1 h at room temperature, followed by an incubation with horseradish peroxidase (HRP)-conjugated secondary antibody for 1 h at 37 °C. After thorough washing with PBST, the plate was incubated with 100 µl of substrate TMB solution for 15–30 min at 37 °C in darkness. Then 50 µl of stop solution was added to terminate color development. The plate was read with a microplate reader at the absorbance of 450 nm. The OD value was calculated using blank control space as zero reference. At the same time, the standard curve was drawn according to the results for the positive control with known concentrations, and then the concentration of each protein was calculated according to the correlation coefficient and the formula of the standard curve.

### Quantitative real-time PCR (qRT-PCR)

As mentioned before, equal numbers of MDA-MB-435s cells, MDA-MB-435s-HM cells, CD133^+^ HPCs, and co-cultured MDA-MB-435s-HM cells and CD133^+^ HPCs (50%:50%) were cultured in serum-free medium for 24 h, and then the cells were collected for total RNA extraction using a kit from Shanghai Fastagen Biotechnology Co., Ltd. (Shanghai, China). The first strand cDNA from 1 µg of total RNA was synthesized using the RevertAid™ First Strand cDNA Synthesis Kit (Thermo Fisher Scientific). qPCR was carried out at 95 °C for 10 min, 40 cycles of 95 °C for 10 s and 60 °C for 60 s on GeneAmp PCR system 9700 PCR equipment. To avoid genomic DNA contamination, control reactions without reverse transcriptase were performed for each primer combination. The cycle threshold (*C*_T_) value for each sample was determined by three biological replicates. The expression of the *GAPDH* gene was used as the internal control. Relative expression of target genes was normalized to that of the reference gene and calculated using the 2^−ΔΔ*CT*^ method. All the primers used for qRT-PCR were listed in Table 1.

**Table 1:** Primers used for qRT-PCR

Gene name	Forward primer	Reverse primer
CXCL-16	GACATGCTTACTCGGGGAT	GGACAGTGATCCTACTGGG
IL-2Rα	GAACACAACGAAACAAGT	GGCTGCATTGGACTTTGCA
IL-2Rγ	TGCATTGGAAGCCGTGGTT	GTTCCCGTGGTATTCAGTA
MMP-1	GGGGCTTTGATGTACCCTA	TGTCACACGCTTTTGGGGTT
MMP-9	GGGACGCAGACATCGTCAT	TCGTCATCGTCGAAATGGG
PDGFR-α	TTGAAGGCAGGCACATTTA	GCGACAAGGTATAATGGCA
SDF-1α	ATTCTCAACACTCCAAACT	ACTTTAGCTTCGGGTCAAT
TGF-α	AGGTCCGAAAACACTGTGA	AGCAAGCGGTTCTTCCCTT
PECAM-1	ACCGTGACGGAATCCTTCT	GCTGGACTCCACTTTGCAC
TIMP-4	CATGTACGTTGCTATCCAG	CTCCTTAATGTCACGCACG

### Cell migration assay

A cell migration assay was carried out using Transwell cell culture chambers (8-µm pore size, EMD Millipore, Billerica, MA, USA). Briefly, MDA-MB-435s-HM cells were serum starved for 24 h and placed in the upper chamber of a 24-well Transwell (5 × 10^4^/well) in serum-free medium. A total of 600 µl of RPMI 1640 complete medium containing specific inhibitors or antibodies to the most significant proteins identified by protein microarray as chemoattractants was added to the lower chamber. The cells were incubated in a 37 °C incubator with 5% CO_2_ for 24 h, and then the filter side of the upper chamber was cleaned with a cotton swab, fixed with 4% paraformaldehyde for 30 min, and stained with 0.1% crystal violet for 20 min. The migrated cells were photographed and counted in five random fields under a light microscope.

### Validation of protein microarray results in vivo

MDA-MB-435s-HM cells were infected with 20 MOI of pGC-FU-Luciferase lentiviruses to obtain a stable cell line and generate a lung metastasis mouse model as described above. Between 10 and 20 days after injection, 0.5 ml of each specific inhibitor or antibody [including TIMP-1 (ProSpec-Tany TechnoGene Ltd., Rehovot, Israel), PDGFR tyrosine kinase inhibitor III (Santa Cruz Biotechnology, Santa Cruz, CA, USA) or anti-human CD31 antibody WM59 (WM59 clone, eBioscience, Thermo Fisher Scientific)] to the most significant proteins identified by protein microarray analysis were intraperitoneally injected every other day at a concentration of 10 µg/ml, while the same volume of saline was injected into control mice. After treatment, six mice in each group were examined based on the bioluminescence of luciferase in the lung area every 4–6 days as described above. Moreover, the ratio of VEGFR1^+^CD133^+^ HPCs in the lung at different time points was examined following the protocol outlined above.

### In vivo invasion of MDA-MB-435s-HM cells

**Fig. 1:**
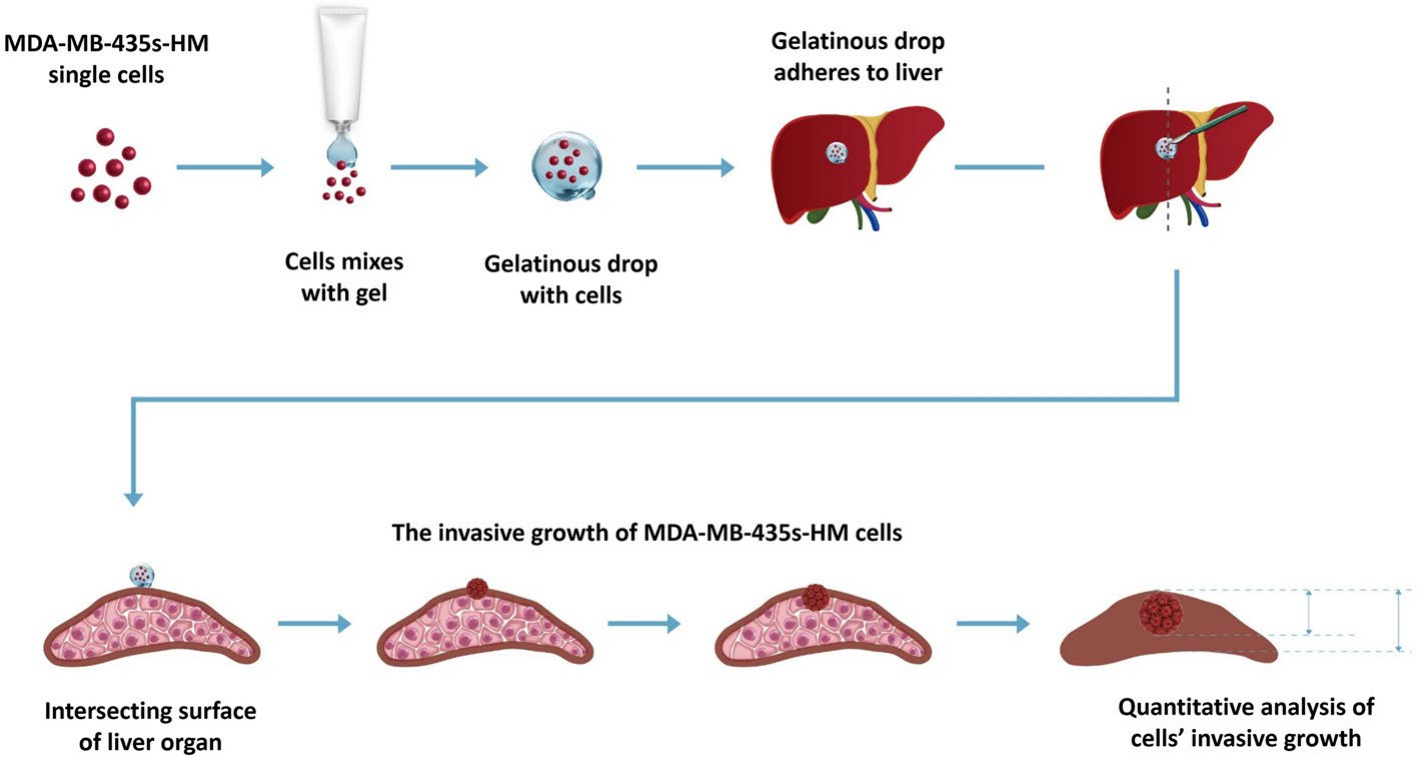
Proposed model for examination of invasive growth of MDA-MB-435s-HM cells. MDA-MB-435s-HM cells were mixed with gel and adhered to the surface of the nude mouse liver. The invasive growth of cells was examined by calculating the thicknesses of tumor nodules or the liver

MDA-MB-435s-HM cells were co-cultured with HPCs. For different experiments, cells were treated with inhibitors of various pathways. Then, cells were mixed with biological–medical gel (Cai-Hong-Yi-Xue-She-Bei Corporation, Kunming City, Yunnan Province, China) and adhered to the surface of the nude mice’s livers (Meng et al. 2018). After 4–8 weeks of growth, mice were screening by ^Micro^PET experiments following methods described by Feng et al. (2018), Xie et al. (2018), and Zhang et al. (2018a). Livers showing nodules formed by the MDA-MB-231 cells were collected for Masson staining, following methods described in our previous work (Meng et al. 2018). Photographs of the Masson staining were quantitatively analyzed to determine the relative invasive growth of the MDA-MB-435s-HM cells using ImageJ software, following methods described in our previous work (Meng et al. 2018): the thicknesses of the nodules or liver were determined, and the relative invasive growth of the MDA-MB-231 cells in the nude mouse liver (the nodule thickness relative to that of the liver organ) was calculated as follows: (nodule thickness in the control group)/(liver thickness in the control group) × 100% or (nodule thickness in the experimental group)/(liver thickness in the experimental group) × 100%. The inhibition rate for each group was calculated as follows: [(relative invasive growth in the control group) − (relative invasive growth in the experimental group)]/(relative invasive growth in the control group) × 100%. The proposed model and flow-process diagram for invasive growth examination are shown in Fig. 1.

### Statistical analysis

The data are presented as mean ± SD. The statistical analyses were performed by one-way analysis of variance (ANOVA) using SPSS17.0 software (SPSS, Inc., Chicago, IL, USA). *P* < 0.05 was considered as statistically significant.

## Results

### Successful establishment of a lung metastasis model in nude mice

**Fig. 2:**
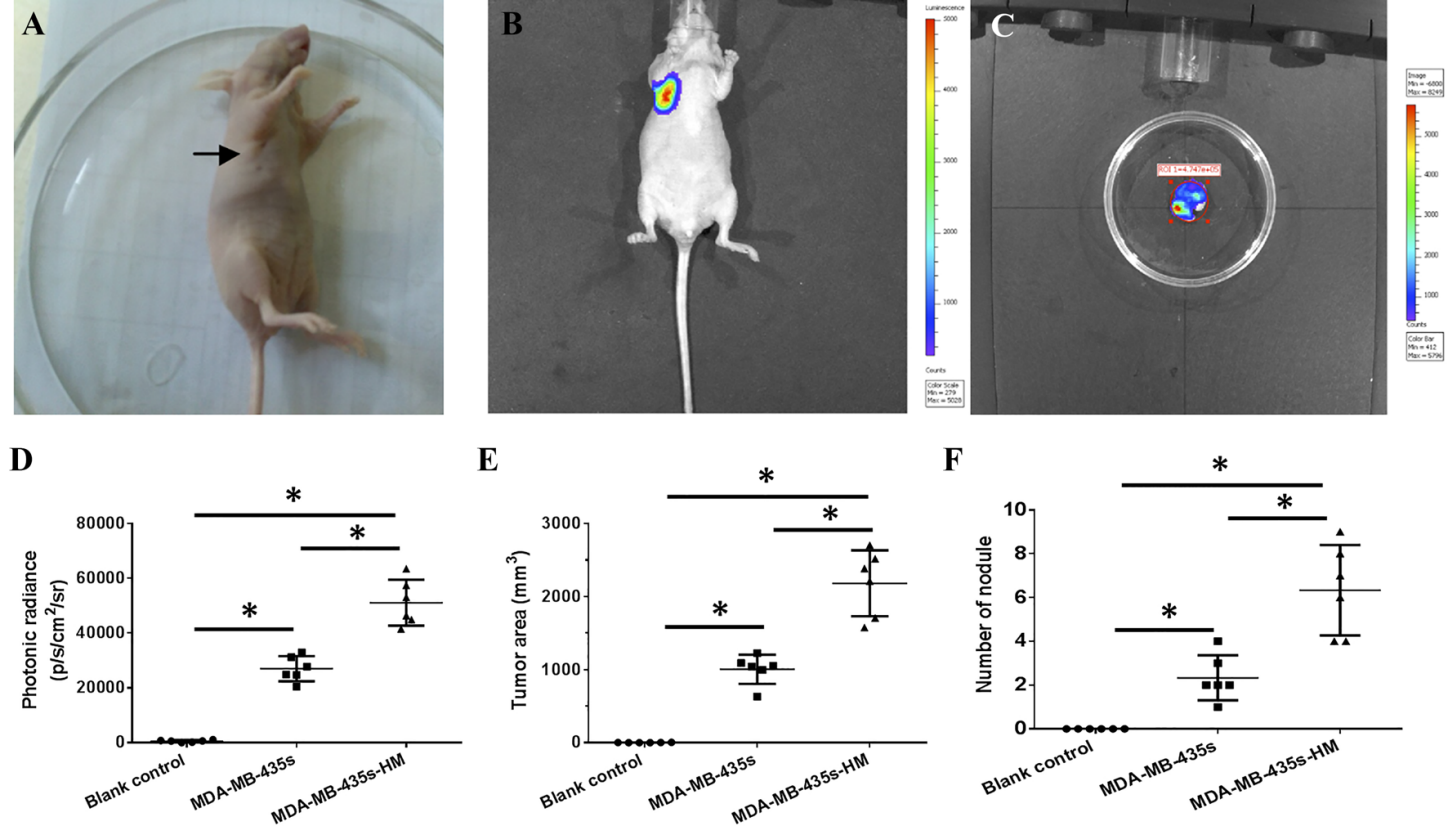
Establishment of a lung metastasis model in nude mice. **a** Representative image of xenografted tumor at 12 days after cells injection. One million cells stably expressing luciferase were injected subcutaneously. A 2–4 mm tumor nodule (indicated by arrow) was observed at 12 days after cell injection. **b** The bioluminescence of luciferase in the xenografted tumor was captured by an in vivo imaging system at 12 days after cell injection. **c** The bioluminescence of luciferase in the lung area was captured under an in vivo imaging system, indicating lung metastasis. Quantitative results for changes in the **d** luciferase intensity, **e** tumor area, and **f** number of tumor nodules are shown as mean ± SD. *P < 0.05

After six rounds of screening, we established the MDA-MB-435s-HM cell line. MDA-MB-435s-HM cells stably expressing luciferase were generated using pGC-FU-luciferase lentiviruses and subcutaneously injected into nude mice. An oval or round tumor node was observed at the injection site at 12 days after injection (Fig. 2a), and strong bioluminescence of luciferase in the tumor node could be observed by in vivo bioluminescence imaging (Fig. 2b). Bioluminescence of luciferase in the lung area could not be detected until 68 days after injection (Fig. 2c). The average time to the detection of bioluminescent luciferase in the lung area was 70.67 ± 2.16 days. Moreover, the intra-lung growth of MDA-MB-435s and MDA-MB-435s-HM cells was assessed based on luciferase intensity (Fig. 1d), tumor volume (Fig. 1e), and number of tumor nodules (Fig. 1f). The intra-lung growth of MDA-MB-435s-HM cells was much greater than that of MDA-MB-435s cells.

### Presence of VEGFR1^+^CD133^+^ HPCs during the progression of lung metastasis

**Fig. 3:**
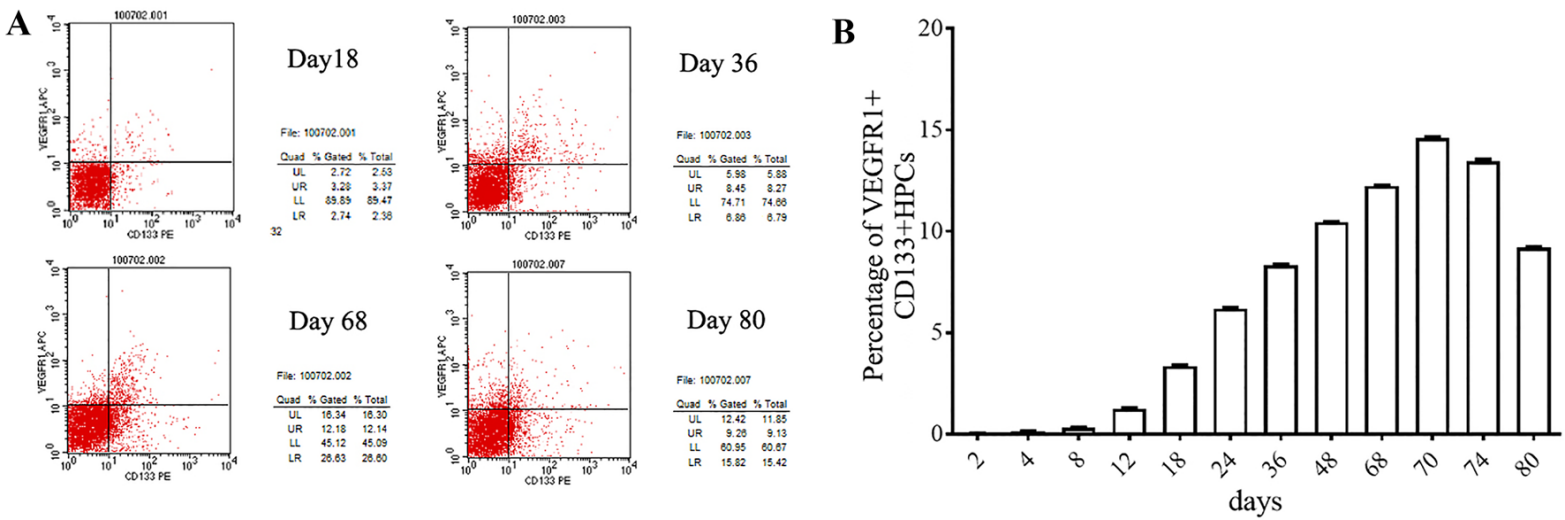
VEGFR1^+^ HPC counts change in the lung during the progression of lung metastasis. **a** Representative flow cytometric graphs for VEGFR^+^ HPCs in the lung at days 18, 36, 68, and 80 after injection. The xenografted mouse models were generated as shown in Fig. 1. At each time point, six mice were killed for evaluation of bioluminescence in the lung area. The lung was dissected to isolate the cells, and the cell suspension was labeled with VEGFR1 and CD133 antibodies. **b** According to the flow cytometric results, the ratio of VEGFR1^+^CD133^+^ HPCs in the lung cell suspension gradually and significantly increased from day 12 (average time to the appearance of skin tumor node) to day 70 (average time to the bioluminescent appearance in the lung), reached a peak (14.6%) on day 70, and significantly decreased from day 70 to 80 (the last time point examined)

Next, we sought to examine the presence of VEGFR1^+^CD133^+^ HPCs during the progression of lung metastasis by flow cytometry. Representative graphs from the flow cytometric analyses of different time points are shown in Fig. 3a. The ratio of VEGFR1^+^CD133^+^ HPCs in the lung cell suspension gradually and significantly increased from day 12 (average time to the appearance of the skin tumor node) to day 70 (average time to bioluminescent appearance in the lung) and reached a peak (14.6%) on day 70 (Fig. 3b). However, the ratio of VEGFR1^+^CD133^+^ HPCs in the lung significantly decreased from day 70 to day 80 (the last time point examined; Fig. 3b) after the metastatic cells appeared in the lung area.

### Effect of HPCs on expression profiles of cytokines/chemokines/MMPs in cancer cells

Previous research demonstrated that VEGFR1^+^ HPCs can form cellular clusters that direct the metastasis of tumor cells to a pre-metastatic niche (Sahoo et al. 2018). Here a similar phenomenon was found in our study. We isolated CD133^+^ HPCs from human UCB and cocultured these cells with MDA-MB-435s cells. Very interestingly, we observed some HPC clusters attached to the floating MDA-MB-435s cells (before 12 h in culture, sFig. 2a), but these HPC clusters were not observed on the surface of adherent MDA-MB-435s cells that attached to the flask after 24 h in culture (sFig. 2b).

**Fig. 4:**
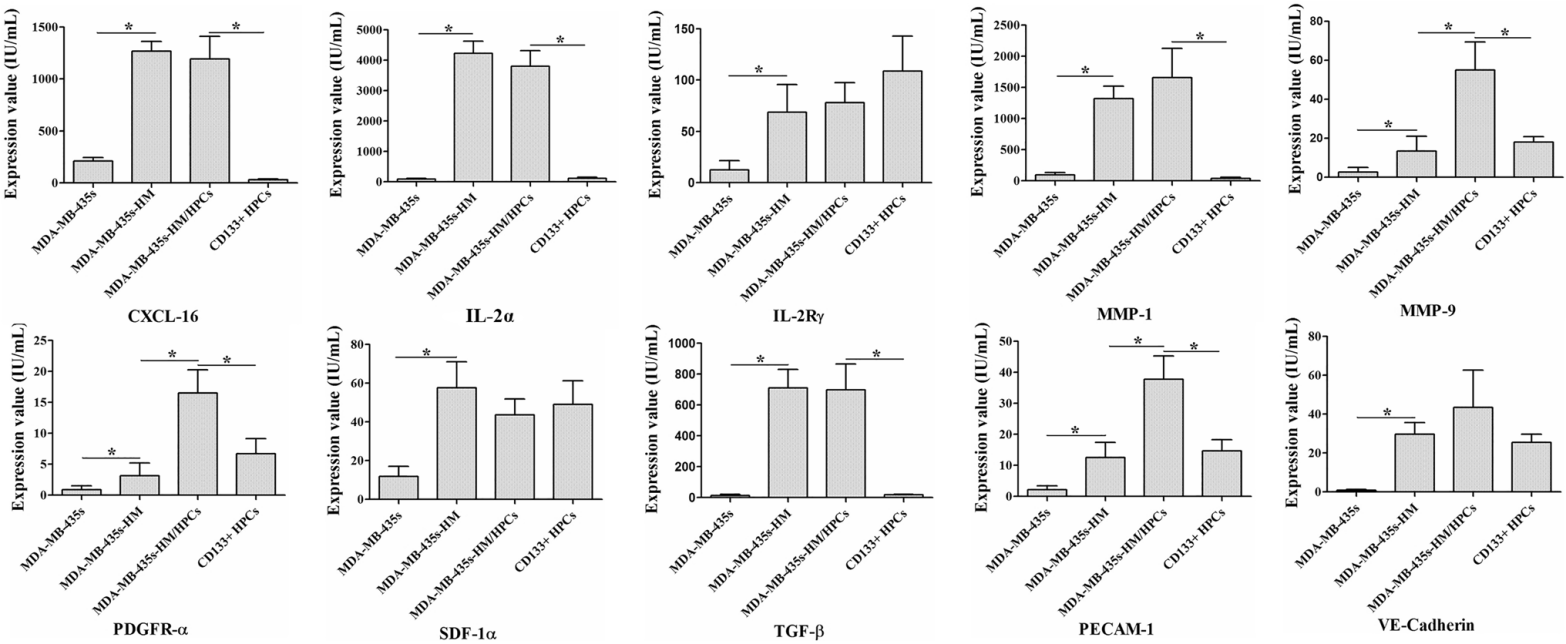
CD133^+^ HPCs affected the expression profiles of cytokines/chemokines/MMPs by cancer cells. HPCs were co-cultured with MDA-MB-435s-HM or MDA-MB-435s cells. The concentrations of CXCL16, IL-2Rα, IL-2Rγ, MMP-1, MMP-9, PDGFR-α, SDF-1α, TGF-β, PECAM-1, and VE-cadherin in the supernatant of MDA-MB-435s-HM cells were significantly higher than those in the supernatant of MDA-MB-435s cells (> fivefold, **a**–**j**). The concentrations of MMP-9, PDGFR-α, and PECAM-1 in the supernatant of MDA-MB-435s-HM cells were significantly elevated after co-culture of MDA-MB-435s-HM cells with CD133^+^ HPCs cells (**e, f, i**). The concentrations of CXCL-16, IL-2Rα, MMP-1, MMP-9, PDGFR-α, and PECAM-1 in the supernatant of CD133^+^ HPCs were elevated when MDA-MB-435s-HM cells were co-cultured with CD133^+^ HPCs (**a, b, d, f, i**)

To explore the underlying mechanisms by which CD133^+^ HPCs affect the behaviors of MDA-MB-435s cells, we collected the supernatant (serum-free) of MDA-MB-435s cells, MDA-MB-435s-HM cells, CD133^+^ HPCs, and MDA-MB-435s-HM cells co-cultured with CD133^+^ HPCs, and then protein microarray analysis was performed to determine the changes in secreted cytokines. The staining graphs of protein chips are shown in sFig.3. The results showed that the levels of CXCL16, IL-2Rα, IL-2Rγ, MMP-1, MMP-9, PDGFR-α, SDF-1α (also known as CXCL12), TGF-β, PECAM-1 (also known as CD31), and VE-cadherin in the supernatant of MDA-MB-435s-HM cells were significantly higher than those in the supernatant of MDA-MB-435s cells (> fivefold, *P* < 0.01, Fig. 4a–j). The levels of MMP-9, PDGFR-α, and PECAM-1 in the supernatant of MDA-MB-435s-HM cells co-cultured with CD133^+^ HPCs were significantly higher than those in the supernatant of MDA-MB-435s-HM cells cultured alone (*P* < 0.01, Fig. 4a–j). Moreover, the levels of CXCL-16, IL-2Rα, MMP-1, PDGFR-α, and PECAM-1 in the supernatant of MDA-MB-435s-HM cells co-cultured with CD133^+^ HPCs were higher than those in the supernatant of CD133^+^ HPCs cultured alone (Fig. 4a–j).

### Validation of protein microarray results by ELISA and qRT-PCR

**Fig. 5:**
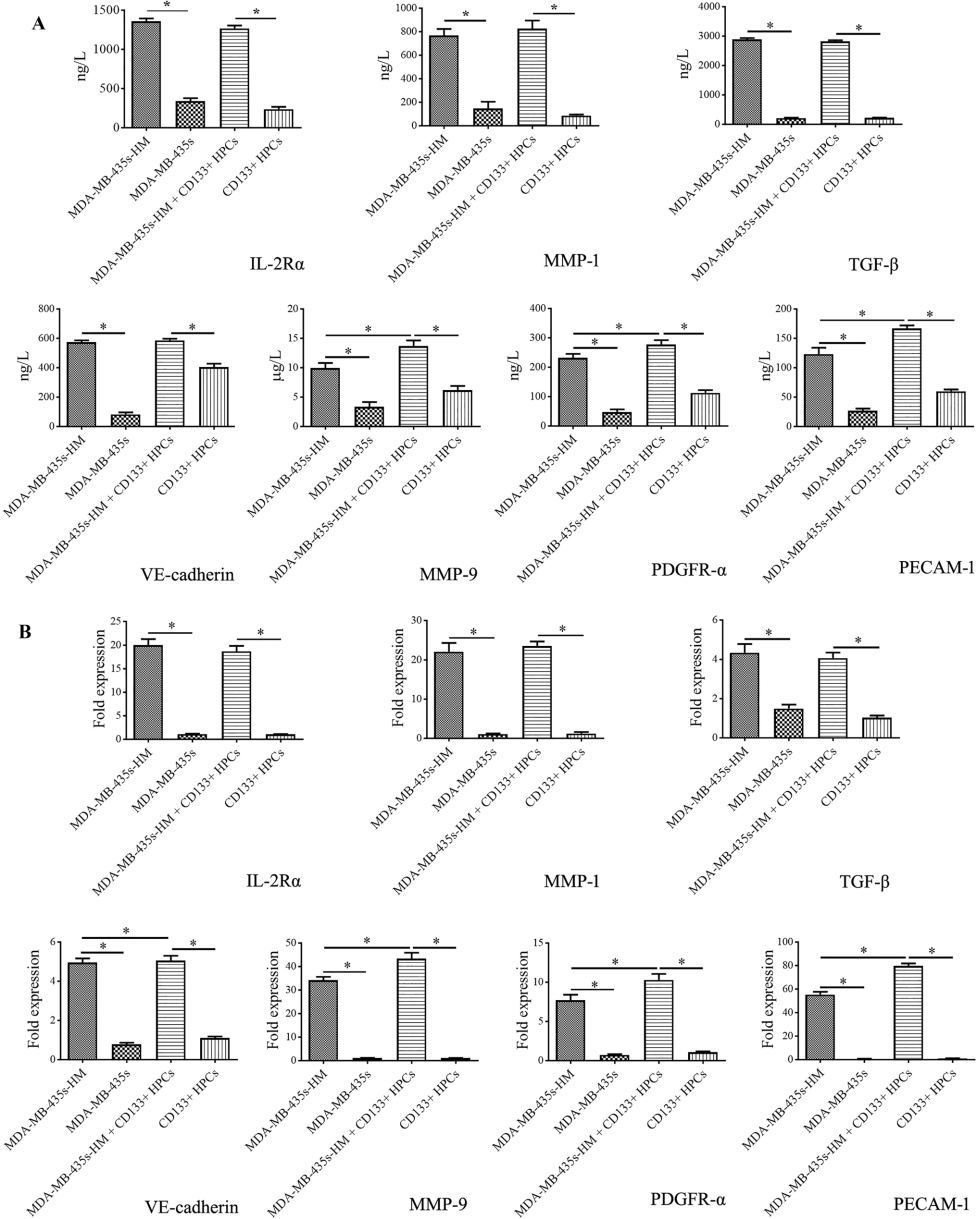
Validation of protein microarray results by ELISA and qRT-PCR. HPCs were co-cultured with MDA-MB-435s-HM or MDA-MB-435s cells. **a** The ELISA results showed trends consistent with those observed by protein microarray in the concentrations of IL-2Rα, MMP-1, MMP-9, PDGFR-α, TGF-β, PECAM-1, and VE-cadherin among the four groups. However, there were no significant differences in CXCL-16, IL-2Rγ and SDF-1α expression among the four groups. **b** The trends in the mRNA expression of IL-2Rα, MMP-1, MMP-9, PDGFR-α, TGF-β, PECAM-1, and VE-cadherin among the four groups were completely consistent with protein concentration results obtained by ELISA

To verify the results of protein microarray analysis, we quantified the levels of CXCL-16, IL-2Rα, IL-2Rγ, MMP-1, MMP-9, PDGFR-α, SDF-1α, TGF-β, PECAM-1 and VE-cadherin in the supernatant by ELISA. Similar to the results of the protein microarray analysis, the concentrations of IL-2Rα, MMP-1, TGF-β, VE-cadherin, MMP-9, PDGFR-α and PECAM-1 in the supernatant of MDA-MA-435s-HM cells and the co-cultured cells were significantly higher than those in the supernatant of MDA-MA-435s cells (*P* < 0.05, Fig. 5a). Moreover, the concentrations of MMP-9, PDGFR-α, and PECAM-1 in the supernatant of the co-cultured cells were significantly higher than those in the supernatant of MDA-MA-435s-HM cells (*P* < 0.05, Fig. 5a). However, the concentrations of CXCL-16, IL-2Rγ, and SDF-1α in the culture medium were not significantly different among the four groups.

To address whether the differential expression of the above cytokines/chemokines/MMPs was affected at the translational or transcriptional level, we further examined the mRNA expression of IL-2Rα, MMP-1, MMP-9, PDGFR-α, TGF-β, PECAM-1, and VE-cadherin in the cells by qRT-PCR. The trends in the mRNA expression of IL-2Rα, MMP-1, MMP-9, PDGFR-α, TGF-β, PECAM-1, and VE-cadherin among the different groups were completely consistent with those in protein concentrations observed by ELISA (Fig. 5b).

### Effect of the inhibition of MMP-9, PDGFR-α, and PECAM-1 on the behaviors and lung metastasis of MDA-MB-435s-HM cells

**Fig. 6:**
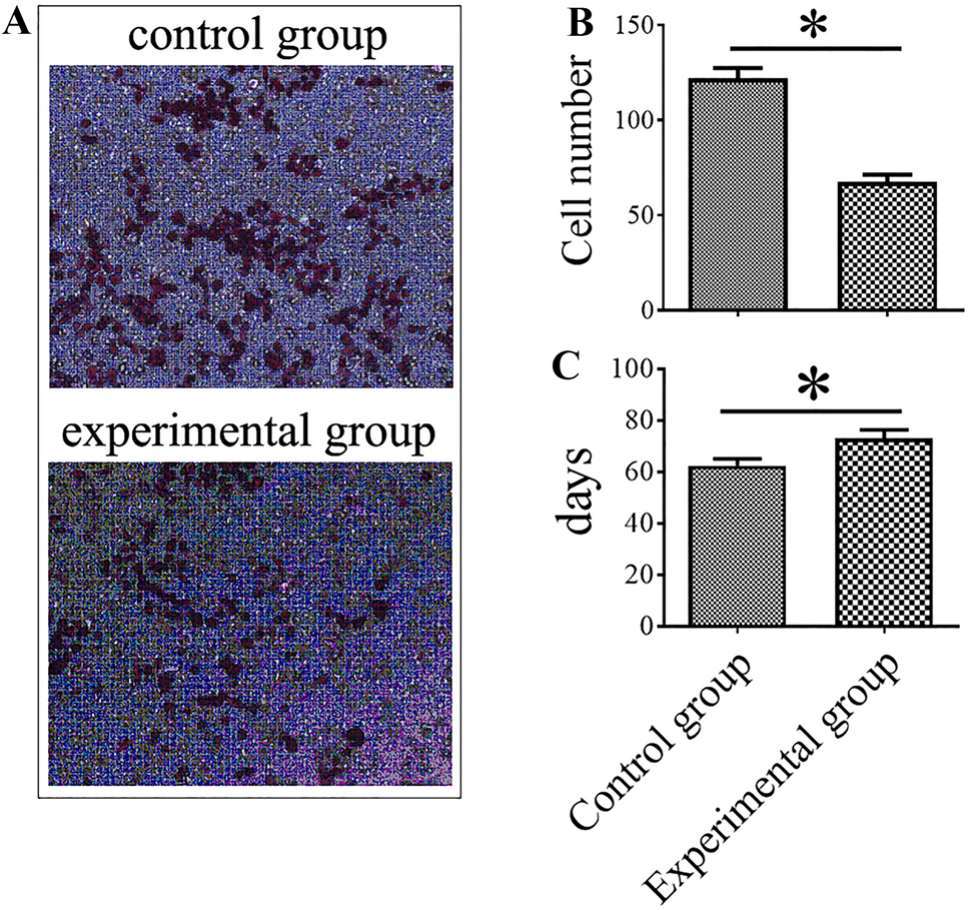
Effect of the inhibition of MMP-9, PDGFR-α, and PECAM-1 on the behaviors of MDA-MB-435s-HM cells. HPCs were co-cultured with MDA-MB-435s-HM cells. **a** Representative images of MDA-MB-435s-HM cell migration. TIMP-1, PDGFR tyrosine kinase inhibitor III, and WM59 were added to the culture medium in the experiment group, and normal saline was added to the control group. A significantly decreased number of migrated cells could be seen in the experimental group. **b** Statistical analysis of cell migration. **c** Inhibition of MMP-9, PDGFR-α, and PECAM-1 suppresses lung metastasis of MDA-MB-435s-HM cells

Because the levels of MMP-9, PDGFR-α, and PECAM-1 in the MDA-MB-435s-HM cells were significantly increased compared with those in the MDA-MB-435s cells and further increased when MDA-MB-435s-HM cells were co-cultured with CD133^+^ HPCs, we predicted that MMP-9, PDGFR-α, and PECAM-1 play important roles in the lung metastasis of MDA-MB-435s cells. To verify this hypothesis, we examined the changes in the behaviors of the cancer cells when MMP-9, PDGFR-α, and PECAM-1 (CD31) were blocked in vitro and in vivo. Tissue inhibitor of metalloproteinase-1 (TIMP-1) has been demonstrated to be a natural inhibitor of MMP-9 (Zhou et al. 2018a; Ligi et al. 2018). PDGFR tyrosine kinase inhibitor III is a cell-permeable, potent, selective, and ATP-competitive inhibitor for PDGFR-α (Abdollahi et al. 2005; Papadopoulos et al. 2018). The anti-human CD31 antibody has been reported to recognize the D2 extracellular portion of CD31 and to block its function (Wu and Lian 1997). In this study, the functions of MMP-9, PDGFR-α. and PECAM-1 in MDA-MB-435s-HM cells were blocked by the combination of TIMP-1, PDGFR tyrosine kinase inhibitor III or anti-human CD31 antibody WM59. The migratory capacity of MDA-MB-435s-HM cells in vitro was significantly reduced after addition of TIMP-1, PDGFR tyrosine kinase inhibitor III (Imatinib mesylate, STI571) and WM59 antibody to the culture medium (Fig. 6a, b). Moreover, no attachment of HPC clusters to the floating MDA-MB-435s-HM cells was observed during the first 24 h after addition of the inhibitor combination to the culture medium (sFig. 2c, d). We further examined the effect of TIMP-1, PDGFR tyrosine kinase inhibitor III, and WM59 antibody on the lung metastasis of MDA-MB-435s-HM cells. In the experimental group, the combination of inhibitors was injected into the abdominal cavity of mice every other day from day 10 after inoculation of MDA-MB-435s-HM cells subcutaneously to the end of the experiment, and in the control group, the same volume of normal saline was injected. The average time to the appearance of detectable lung metastasis in the control group was 62.33 ± 2.80 days, while in the experimental group, the time was extended to 73.0 ± 3.35 days (*P* < 0.05, Fig. 6c).

### Effect of the inhibition of MMP-9, PDGFR-α, and PECAM-1 on the recruitment of VEGFR1^+^ HPCs to the lung

**Fig. 7:**
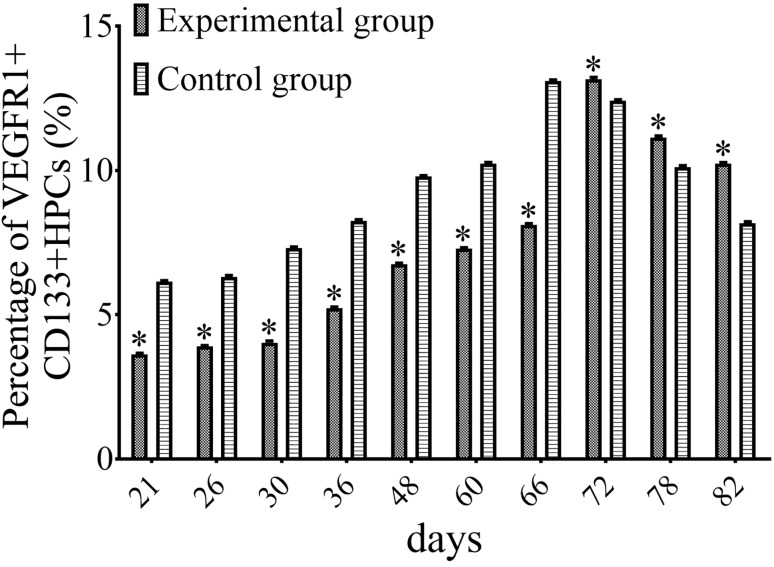
Inhibition of MMP-9, PDGFR-α, and PECAM-1 decreases the amount of VEGFR1^+^CD133^+^ HPCs in the lung. The peak of the average ratio of VEGFR1^+^ HPCs was postponed and synchronous to the appearance of detectable lung metastasis. Transfection of miR-140-3p enhanced the antitumor effect of sorafenib on MHCC97-H cells’ intrahepatic growth

Our results demonstrated that the recruitment of VEGFR1^+^ HPCs to the lung was gradually increased during the progression of lung metastasis of MDA-MB-435s cells. Moreover, the secretion of MMP-9, PDGFR-α, and PECAM-1 by MDA-MB-435s-HM cells was dramatically increased when these cells were co-cultured with CD133^+^ cells, and the inhibition of MMP-9, PDGFR-α, and PECAM-1 could inhibit the migration and lung metastasis of MDA-MB-435s-HM cells. Therefore, we speculated that the inhibition of MMP-9, PDGFR-α, and PECAM-1 might influence the recruitment of MDA-MB-435s-HM cells to the lung. Indeed, compared with that in the control group, the average ratio of VEGFR1^+^ HPCs in the lung of the experimental group (treated with TIMP-1, PDGFR tyrosine kinase inhibitor III, and WM59 antibody) was significantly decreased from day 21 to day 66 (*P* < 0.05, Fig. 7). However, from day 72 to day 82, the average ratio of VEGFR1^+^ HPCs in the lung of the experimental group was significantly higher than that in the lung of the control group (*P* < 0.05, Fig. 7). The peak in the average ratio of VEGFR1^+^ HPCs was postponed from day 66 to day 72, which was synchronous to the appearance of detectable lung metastasis.

### HPCs enhanced the invasive growth of MDA-MB-435s-HM cells in the nude mouse liver

**Fig. 8:**
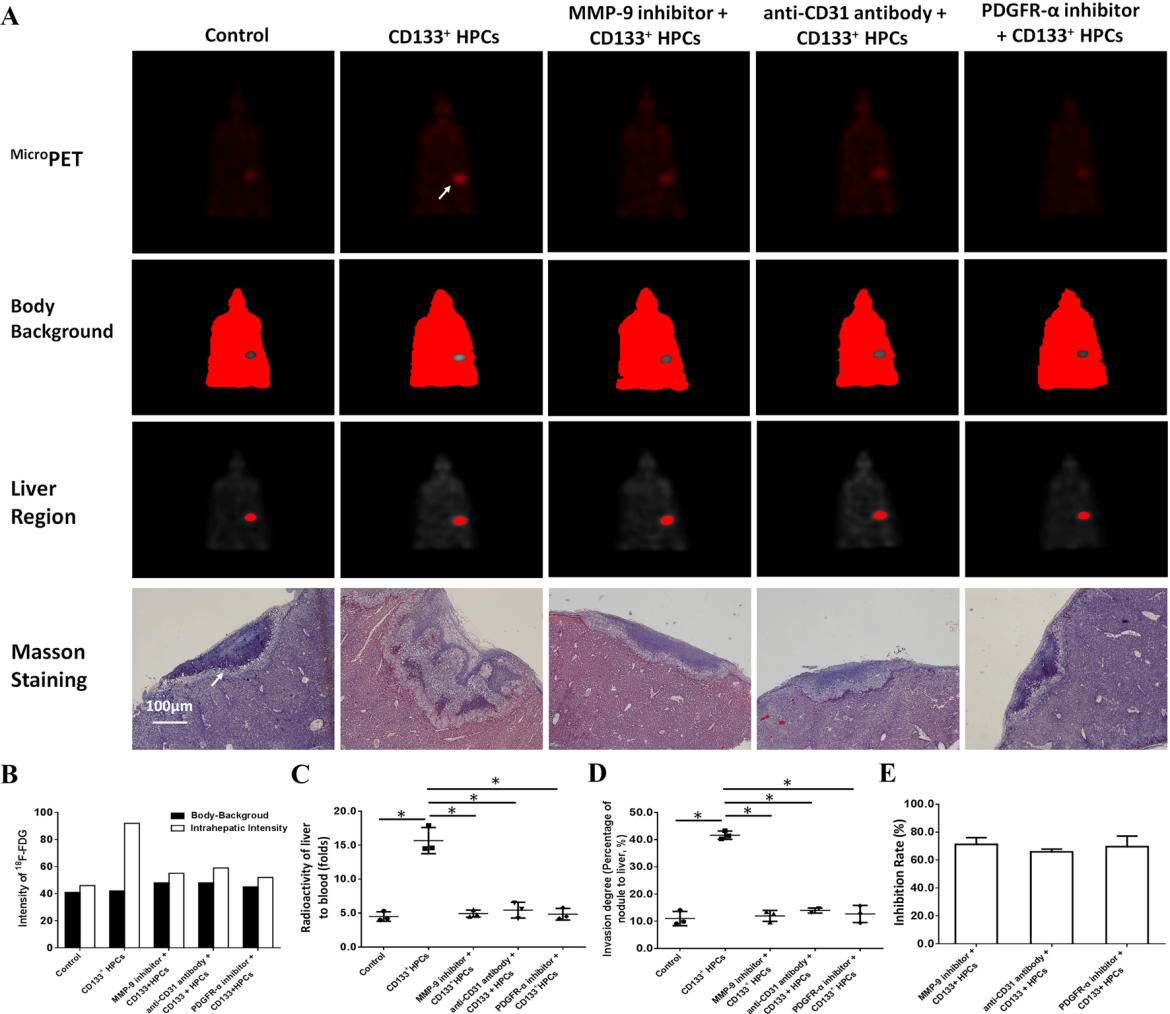
HPCs enhance the invasive growth of MDA-MB-435s-HM cells. HPCs were co-cultured with MDA-MB-435s-HM cells, and cells were treated with an inhibitor of MMP-9, anti-CD31 antibody, or an inhibitor of PDGFR-α. The invasive growth was assessed based on **a**^Micro^PET photographs, area of nodules in liver formed by MDA-MB-435s-HM cells or Masson staining indicating the invasive growth of cells. Quantitative results from **b**^Micro^PET photographs; **c** fold difference in radioactivity between liver and blood; **d** inhibitory rates of inhibitors on ^18^F-FDG intensity or **e** invasive growth of cells. Definitions: inhibitor of MMP-9, tissue inhibitor of metalloproteinase-1 (TIMP-1) shown to be a natural inhibitor of MMP-9; anti-CD31 antibody, anti-human CD31 antibody recognizing the D2 extracellular portion of CD31 to block its function; inhibitor for PDGFR-α, PDGFR tyrosine kinase inhibitor III shown to be an ATP-competitive inhibitor of PDGFR-α. *P < 0.05. White arrow in Figure indicates the nodules in mouse liver

**Fig. 9:**
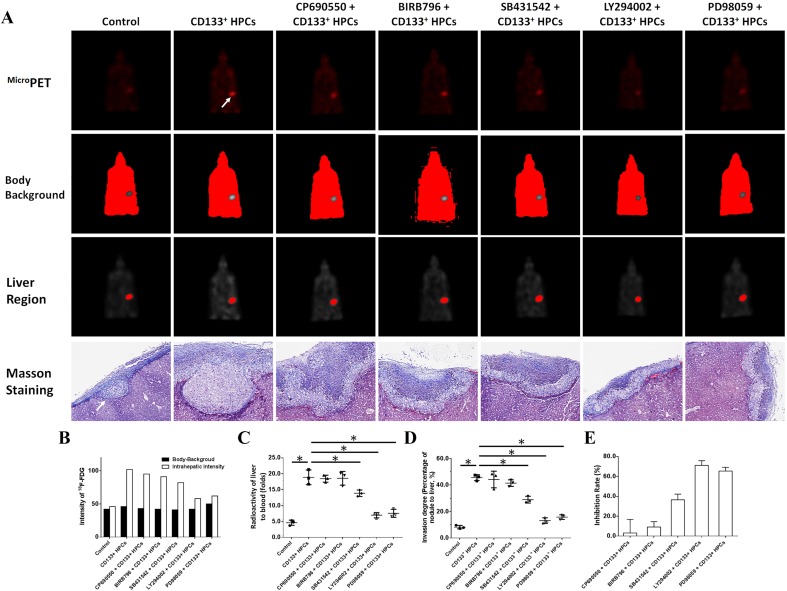
HPCs enhances the invasive growth of MDA-MB-435s-HM cells. HPCs were co-cultured with MDA-MB-435s-HM cells, and cells were treated with an inhibitor of LY-294002, PD98059, SB431542, CP690550, or BIRB796. The invasive growth was assessed based on **a**^Micro^PET photographs, as area of nodules in liver formed by MDA-MB-435s-HM cells or Masson staining indicating the invasive growth of cells. Quantitative results from **b**^Micro^PET photographs; **c** fold difference in radioactivity between liver and blood; **d** inhibitory rates of inhibitors on ^18^F-FDG intensity or **e** invasive growth of cells. Definitions: inhibitor of MMP-9, tissue inhibitor of metalloproteinase-1 (TIMP-1) shown to be a natural inhibitor of MMP-9; anti-CD31 antibody, anti-human CD31 antibody recognizing the D2 extracellular portion of CD31 to block its function; inhibitor for PDGFR-α, PDGFR tyrosine kinase inhibitor III shown to be an ATP-competitive inhibitor of PDGFR-α. *P < 0.05. White arrow in Figure indicates the nodules in mouse liver

Transplantation of MDA-MB-435s-HM cells into the nude mouse liver was used as a mouse model to mimic the in vivo invasive growth of cells. As shown in Figs. 8 and 9, MDA-MB-435s-HM cells penetrated the surface of the liver, destroyed the tissue and invasively grew into the liver. Moreover, co-culture with HPCs significantly enhanced the invasive growth of MDA-MB-435s-HM cells in the liver, and treatment of TIMP-1, PDGFR tyrosine kinase inhibitor III, or anti-CD31 antibody significantly inhibited the invasive growth of MDA-MB-534s-HM cells induced by HPCs (Fig. 8). Moreover, treatment with LY-294002 (a specific inhibitor of the PI3K/AKT pathway) or PD98059 (a specific inhibitor of the MEK/ERK pathway) but not BIRB796 (a specific inhibitor of the p38 MAPK pathway) or CP690550 (a specific inhibitor of the Jak/STAT pathway) inhibited the invasive growth of the cells. The inhibitory effect of SB431542 (a specific inhibitor of the TGFβ/Smad pathway) was much weaker than that of LY-294002 or PD98059. Therefore, HPCs could enhance the invasive growth of MDA-MB-435s-HM cells by enhancing the expression of MMP, PDGFR or CD31, and the effect of HPCs was mainly mediated via the PI3K/AKT or MEK/ERK pathways.

## Discussion

Metastasis is the leading cause of death among melanoma cases. In recent decades, the tumor microenvironment has attracted more and more attention and researchers have achieved significant progress in this field. It has been demonstrated that the tumor-specific ‘pre-metastatic niche’ is a key driving force for tumor metastasis, and this has been confirmed in mouse models of cancer metastasis by some groups (Zingg et al. 2018). However, the key factors for the formation of the pre-metastatic niche remain unclear. In the present study, we successfully used MDA-MB-435s cells stably expressing luciferase to establish a mouse model of lung metastasis and investigated the molecular factors that affect the formation of metastatic colonies in the lung. The invasion of breast cancer cells was evaluated using several models. MDA-MB-435s-HM cells, which had been co-cultured with HPCs, were mixed with biological gels and adhered to the liver to mimic breast cancer to liver metastasis. Cells were treated with inhibitors for inhibition of MMP-9, PDGFR-a, and PECAM-1. The infiltrative growth of cells in the liver reflected the invasive growth of MDA-MB-435s-HM cells in the liver. The results were quantitatively analyzed and the inhibitory activities of the inhibitors on invasive MDA-MB-435s-HM cell growth were examined. Highly aggressive growth is the foremost feature of human malignancy tumors, e.g., triple negative breast cancer. The invasion or migration of cancer cells is often measured by in vitro or in vivo models. Although Transwell experiments are commonly used to examine the invasion or migration of human cancers, results from Transwell experiments cannot easily mimic the invasion or migration of cancer cells in vivo. To resolve this problem, MDA-MB-435s and MDA-MB-435s-HM cells were injected into the lateral tail vein to mimic breast cancer to liver metastasis. Although our results reflected the lung-metastasis of cells, the massive capillary system in the lung would cause some deposition of tumor cells in the lung, and the pathological metastasis of breast cancer cells cannot be simply mimicked by these methods. Therefore, our work also attempted to measure the invasive growth of cells within the liver. The intrahepatic tumor model that mimics breast cancer to liver metastasis is more representative compared with the regular tumor metastasis model and could be a useful tool to evaluate the therapeutic efficiency of anti-tumor therapies.

The recruitment of non-resident cells such as BMDCs is a key step in the formation of the pre-metastasis niche (Sahoo et al. 2018). They observed β-galactosidase-positive (β-gal+) and green fluorescent protein-positive (GFP^+^) BMDCs colonized at tumor-specific pre-metastatic sites before the arrival of tumor cells using an in vivo imaging system (IVIS) and further demonstrated that BM-derived VEGFR1^+^ HPCs play a dominant role in forming pre-metastatic niches for tumor metastasis (Sahoo et al. 2018). Some further studies also demonstrated the role of BMDCs or HPCs in forming pre-metastatic niches (Lei et al. 2018; Kaplan et al. 2007). In the present study, we established a mouse model of lung metastasis using luciferase-expressing MDA-MB-435s cells and an IVIS. Unlike previous studies, we did not infuse exogenous BMDCs into the mouse model of tumor metastasis, but we did observe increasing recruitment of endogenous VEGFR1^+^CD133^+^ HPCs to the lung during the progression of lung metastasis and a peak in this recruitment on day 70 after injection. Interestingly, the recruitment of VEGFR1^+^CD133^+^ HPCs in the lung was gradually decreased once lung metastasis was established. These results further verify the previous finding that VEGFR1^+^ HPCs can initiate and maintain the pre-metastatic niche in the lung.

Previous research demonstrated that in addition to VEGFR1^+^ cellular clusters, tumor-specific cytokines/chemokines also play important roles in the multidimensional program driving metastatic spread (Sahoo et al. 2018). In this study, we observed higher levels of CXCL-16, IL-2Rα, IL-2Rγ, MMP-1, MMP-9, PDGFR-α, SDF-1α, TGF-β, PECAM-1, and VE-cadherin in the culture medium of MDA-MB-435s-HM (highly metastatic) cells (by protein microarray and ELISA) and higher expression of their transcripts in the MDA-MB-435s-HM cells, compared to levels in the parental MDA-MB-435s cells. The functions of these proteins in tumor metastasis have been supported by many studies. It has been demonstrated that the CXCL-16/CXCR6 axis promotes hepatocellular carcinoma invasiveness and induces a proinflammatory tumor environment for metastasis in various cancer types (Gao et al. 2012; Richardsen et al. 2015). The level of IL-2Rα in the serum of early gastric cancer patients may be a good predictor for lymph node metastasis (Baik et al. 2012; He et al. 2017). MMP-9 is one of the most important MMPs involved in tumor invasion and metastasis (Si et al. 2018), whereas MMP-1 has also been reported to activate endothelial PAR-1 to facilitate endothelial permeability and transendothelial migration of tumor cells, and thus to promote metastatic dissemination (Andrae et al. 2008). Both the PDGF/PDGFR and SDF-1/CXCR4 signaling pathways are involved in mediating EMT and angiogenesis, and thus, the regulation of tumor growth, invasion, and metastasis (Petit et al. 2007; Stumpf et al. 2018; Hu et al. 2014; Lange et al. 2018; Peng et al. 2017), but this is the first time the secretion of PDGFR-α (by protein microarray and ELISA) from cancer cells has been detected. TGF-β is a key regulator of tumor progression, and high TGF-β expression has been observed in many cancer types (Vander Ark et al. 2018; Watt et al. 2018; Yang et al. 2018). PECAM-1/CD31 is a transmembrane glycoprotein involved in cell adhesion and a common biomarker for endothelial cells (Basilio-de-Oliveira and Pannain 2015; Cao et al. 2018). High CD31 expression is often found in tumor tissues by immunohistochemistry and associated with a poor prognosis among cancer patients (Basilio-de-Oliveira and Pannain 2015; Cao et al. 2018), but high CD31 secretion by tumor cells has rarely been analyzed by protein microarray or ELISA, as observed in this study. VE-cadherin, also known as cadherin-5, is an adhesion molecule uniquely expressed in endothelial cells that has been used to assess microvessels and angiogenesis in human breast cancer (Zhao et al. 2018; Tang et al. 2018; Zhou et al. 2018). High expression of VE-cadherin in the tumor correlates with poor prognosis in human gastric cancer (Higuchi et al. 2017), but the level of soluble VE-cadherin has not been detected in cancer cells or tissues. A previous study reported that a high level of soluble VE-cadherin is associated with a poor outcome of severe sepsis (Han et al. 2018). Moreover, there are close interactions among these proteins during tumor progression, which has been confirmed in different cancers (Liu et al. 2014; Moore-Smith et al. 2017). Although we did not obtain mechanistic data for the high expression of these proteins in MDA-MB-435s-HM cells, this cell line was established from lung colonies of metastatic MDA-MB-435s cells after six rounds of screening. Compared to those of the parental MDA-MB-435s cells, the properties of MDA-MB-435s-HM cells were largely affected by many systematic factors in the tumor microenvironment. Considering these findings together with the results of our previous studies, it is reasonable to speculate that these proteins contribute to the metastatic potential of MDA-MB-435s-HM cells.

The formation of the pre-metastatic niche is a complex process affected by tumor-secreted factors and tumor-shed extracellular vehicles (EVs); however, the cellular and molecular events that occur during the formation of the pre-metastatic niche have not been elucidated. One of the important functions of the pre-metastatic niche is to attract circulating tumor cells to the pre-metastatic site. Here we did observe adhesion between CD133^+^ HPCs and MDA-MB-435s cells, but the adhesion disappeared after attachment of the tumor cells to the culture flask. Moreover, we observed further elevation of the levels of cell adhesion-associated molecules such as VE-adhesion and PECAM-1 during co-culture of CD133^+^ HPCs and MDA-MB-435s-HM cells. Recent studies have attempted to uncover the molecular mechanisms responsible for the settling of circulating tumor cells at pre-metastatic sites. Kaplan et al. demonstrated that VEGFR1 and VLA-4 are involved in the establishment of the pre-metastatic niche and communication between HPCs and tumor cells (Sahoo et al. 2018). S100A4 has been shown to attract T cells to the primary tumor and the pre-metastatic niche (Grum-Schwensen et al. 2015). Cytochrome P450 (CYP) 4A in tumor-associated macrophages can promote the formation of a pre-metastatic niche (evidenced by the recruitment of VEGFR1^+^ myeloid cells) and further affect metastasis, and the combined blocking of TGF-β, VEGF, and SDF-1 can suppress VEGFR1^+^ myeloid cell migration and fibroblast activation induced by CYP4A (Zhao et al. 2016; Chen et al. 2017). In this study, we found that co-culture of MDA-MB-435s-HM cells and CD133^+^ HPCs further stimulated the secretion of MMP-9, PDGFR-α, and PECAM-1 compared with levels secreted by MDA-MB-435s-HM cells. More importantly, the combined blockage of MMP-9, PDGFR-α, and PECAM-1 could significantly suppress cell migration and lung metastasis of MDA-MB-435s-HM cells as well as the recruitment of VEGFR1^+^CD133^+^ HPCs to the lung during the lung metastasis of MDA-MB-435s-HM cells. Moreover, the peak of VEGFR1^+^ HPC recruitment was postponed from day 66 to day 72, which was synchronous to the appearance of detectable lung metastasis. These data provide solid evidence that MMP-9, PDGFR-α, and PECAM-1 play critical roles in the formation of the pre-metastatic niche and the metastatic potential of MDA-MA-435s-HM cells, which also adds new evidence for the molecular mechanisms of pre-metastatic niche formation and lung metastasis. Additionally, in our further study, it will be interesting to determine the individual roles of MMP-9, PDGFR-α, and PECAM-1 during lung metastasis as well as to learn whether the functions of MMP-9, PDGFR-α, or PECAM-1 are tumor-type or cancer cell-type specific. Moreover, connexins are also important regulators of cancer cell metastasis (Zibara et al. 2015; Ghattass et al. 2014).

Our mechanistic data revealed that inhibition of MMP-9, CD31 or PDGFR could significantly inhibit the invasive growth of MDA-MB-435s-HM cells. However, usage of a single inhibitor could not completely block the effect of HPCs on MDA-MB-435s-HM. These results suggest that MMP-9, CD31 or PDGFR participate in the regulation of cancer cell metastasis via related pathways or compensatory mechanisms. Furthermore, the potential mechanisms, e.g., signaling pathways, mediate the effect of HPCs on MDA-MB-435s-HM cells. Our results showed that treatment with LY294002 or PD98059 but not with BIRB796 or CP690550 could significantly inhibit the effect of HPCs on MDA-MB-435s-HM cells. It is well known that the PI3K/AKT and MEK/ERK pathways play important roles in regulating cancer cell invasion or migration (Chang et al. 2018; Bollaert et al. 2018; Zhang et al. 2018b; Shults et al. 2018; Yan et al. 2018; Johnsen et al. 2018). The Jak/STAT and p38 MAPK pathways mainly regulate the cellular stress response, immune processes, and inflammation (O’Reilly et al. 2018; Ajibade et al. 2012). Interestingly, SB431542 (a specific inhibitor of the TGFβ/Smad pathway) only weakly inhibited the effect of HPCs. The TGFβ/Smad pathway is one of the key regulators of the tissue microenvironment or EMT process (Meyer-Schaller et al. 2018; Fabregat and Caballero-Díaz 2018). Thus, research is urgently needed to determine the exact roles of the TGFβ/Smad pathway in HPCs and MDA-MB-435s-HM cells.

Moreover, HPCs can be obtained from adult bone marrow or UCB (Świerzko et al. 2018; Matsuoka et al. 2018), and there are no differences in the HPCs from different origins. The amount of HPCs is much higher in UCB than in BM, and isolation of HPCs from BM also requires an invasive procedure (Świerzko et al. 2018; Matsuoka et al. 2018). Therefore, we chose to use HPCs from UCB in our manuscript.

In conclusion, we observed increasing recruitment of VEGFR1^+^CD133^+^ HPCs to the lung during tumor metastasis and found high expression of CXCL-16, IL-2Rα, IL-2Rγ, MMP-1, MMP-9, PDGFR-α, SDF-1α, TGF-β, PECAM-1, and VE-cadherin in highly metastatic MDA-MB-435s cells. We further demonstrated that MMP-9, PDGFR-α, and PECAM-1 play dominant roles in the recruitment of VEGFR1^+^CD133^+^ HPCs to the lung and the metastatic potential of MDA-MB-435s-HM cells. These data add new information about the molecular mechanisms for pre-metastatic niche formation and lung metastasis of melanoma, which may provide new therapeutic insights for the treatment of tumor metastasis.

## Electronic supplementary material

Below is the link to the electronic supplementary material.

Supplementary material 1 (TIF 755 KB)

Supplementary material 2 (TIF 1924 KB)

Supplementary material 3 (TIF 1342 KB)

Supplementary material 4 (DOCX 79 KB)
