# Supplementary figures and images for: Effects of VEGFR1+ hematopoietic progenitor cells on pre-metastatic niche formation and in vivo metastasis of breast cancer cells
Source: J Cancer Res Clin Oncol. 2018 Nov 27;145(2):411–27. doi: 10.1007/s00432-018-2802-6 (PMC6373264; doi:10.1007/s00432-018-2802-6)

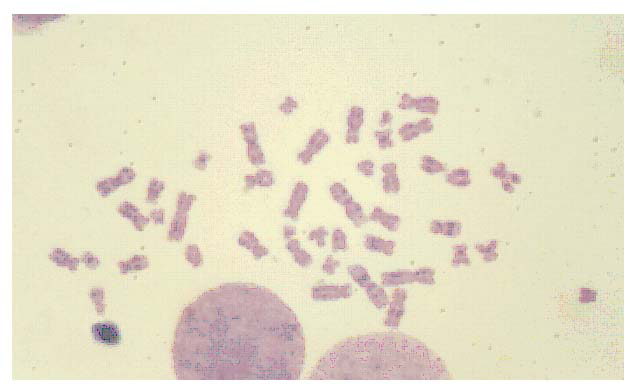

Supplement: Supplementary file 1 — Supplementary material 1 (TIF 755 KB) [file 432_2018_2802_MOESM1_ESM.tif]

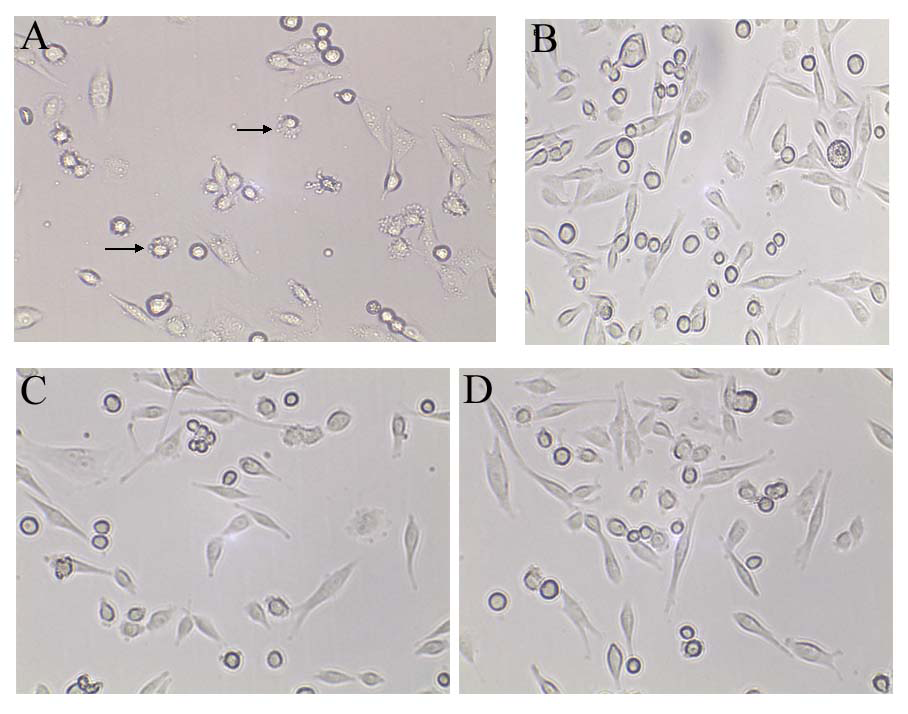

Supplement: Supplementary file 2 — Supplementary material 2 (TIF 1924 KB) [file 432_2018_2802_MOESM2_ESM.tif]

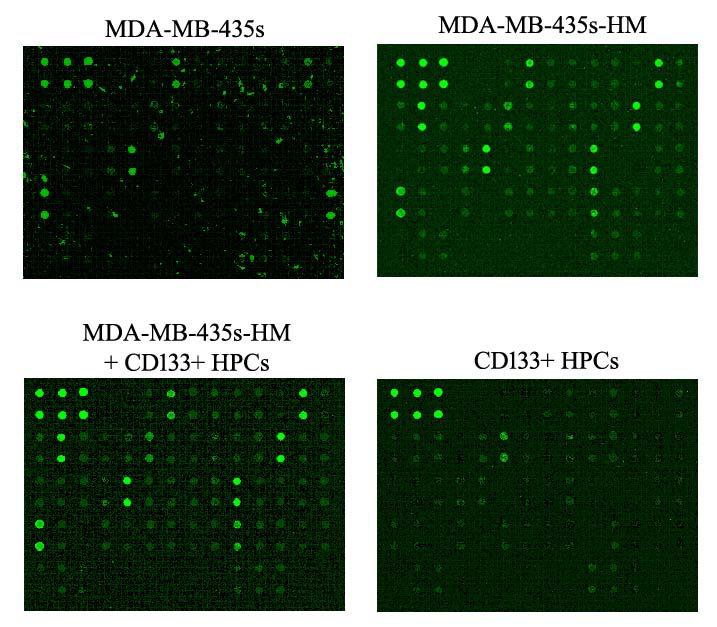

Supplement: Supplementary file 3 — Supplementary material 3 (TIF 1342 KB) [file 432_2018_2802_MOESM3_ESM.tif]
