# Supplementary material for: Effects of VEGFR1+ hematopoietic progenitor cells on pre-metastatic niche formation and in vivo metastasis of breast cancer cells
Source: J Cancer Res Clin Oncol. 2018 Nov 27;145(2):411–27. doi: 10.1007/s00432-018-2802-6 (PMC6373264; doi:10.1007/s00432-018-2802-6)
Supplement: Supplementary file 4 — Supplementary material 4 (DOCX 79 KB) [file 432_2018_2802_MOESM4_ESM.docx]

Supplemental table1 Upregulated and downregulated proteins (between 2-fold and 5-fold) in MDA-MB-435s HM cells in protein microarrays

|  | Protein name | Up/downregulated | fold |
| --- | --- | --- | --- |
| MDA-MB-435s HM cells  *v.s.*  MDA-MB-435s cells | VEGF R2 | Upregulated | 2.061003124 |
|  | IP-10 | Upregulated | 2.078376681 |
|  | MPIF-1 | Upregulated | 2.097234416 |
|  | Activin A | Upregulated | 2.275722452 |
|  | IL-18 BPalpha | Upregulated | 2.328358209 |
|  | IL-5 Ralpha | Upregulated | 2.364738806 |
|  | M-CSF R | Upregulated | 2.375438982 |
|  | Prolactin | Upregulated | 2.375438982 |
|  | POS | Upregulated | 2.399698732 |
|  | VEGF R3 | Upregulated | 2.413135013 |
|  | Siglec-5 | Upregulated | 2.418818072 |
|  | PDGF-AB | Upregulated | 2.422415362 |
|  | BMP-5 | Upregulated | 2.528749694 |
|  | NGF R | Upregulated | 2.529641512 |
|  | SCF R | Upregulated | 2.619402985 |
|  | IL-10 Rbeta | Upregulated | 2.689000649 |
|  | IGF-II | Upregulated | 2.721751719 |
|  | DR6 (TNFRSF21) | Upregulated | 2.781784674 |
|  | PDGF Ralpha | Upregulated | 3.00 |
|  | B7-1(CD80) | Upregulated | 3.089552239 |
|  | IL-9 | Upregulated | 3.141153986 |
|  | IL-2 Rbeta | Upregulated | 3.160859663 |
|  | L-Selectin | Upregulated | 3.333249294 |
|  | CXCL- 16 | Upregulated | 3.640006489 |
|  | LIF | Upregulated | 3.943867618 |
|  | Endoglin | Upregulated | 4.825216025 |
|  | CD14 | downregulated | 0.42638484 |
|  |  |  |  |
| MDA-MB-435s HM cells  *v.s.*  MDA-MB-435s cells/HPCs | LIF | Upregulated | 2.218522771 |
|  | PDGF Ralpha | downregulated | 0.462686567 |
|  |  |  |  |
| MDA-MB-435s HM cells  *v.s.*  MDA-MB-435s cells/HPCs | MMP-13 | Upregulated | 2.382222222 |
|  | Leptin R | Upregulated | 2.655062945 |
|  | PECAM-1 | Upregulated | 3.195977011 |
|  | MMP-9 | Upregulated | 4.038825032 |
|  | ErbB3 | downregulated | 0.364772181 |
